# Differentiating Early Alzheimer’s Disease from MCI Using Comprehensive Semiquantitative Parameters in Dual-Phase Amyloid PET: A Pilot Study

**DOI:** 10.3390/medicina62030529

**Published:** 2026-03-12

**Authors:** Hyung Jin Choi, Ara Cho, Joung Hyun You, Seungchan Park, Suk Hyun Lee, Do Hoon Kim

**Affiliations:** 1 Department of Nuclear Medicine, Chuncheon Sacred Heart Hospital, College of Medicine, Hallym University, 77 Sakju-ro, Chuncheon-si 24253, Gangwon-do, Republic of Korea; 2 Mind-Neuromodulation Laboratory, College of Medicine, Hallym University, Chuncheon-si 24252, Gangwon-do, Republic of Korea; 3 Department of Radiology, Hallym University Kangnam Sacred Heart Hospital, College of Medicine, Hallym University, Seoul 07441, Republic of Korea; 4 Department of Psychiatry, Hallym University Chuncheon Sacred Heart Hospital, College of Medicine, Hallym University, 77 Sakju-ro, Chuncheon-si 24253, Gangwon-do, Republic of Korea

**Keywords:** Alzheimer’s disease, mild cognitive impairment, amyloid PET, dual-phase PET/CT, SUV_diff_

## Abstract

*Background and Objectives*: Dual-phase amyloid PET imaging has been proposed to provide complementary information regarding amyloid burden and cerebral perfusion. This exploratory pilot study evaluated whether semiquantitative parameters derived from dual-phase PET/CT could differentiate individuals operationally classified as Alzheimer’s disease with mild functional impairment (AD-MFI) from those with mild cognitive impairment (MCI). *Materials and Methods*: Twenty-four participants (AD-MFI, *n* = 19; MCI, *n* = 5) underwent dual-phase amyloid PET/CT and structural MRI. Early phase SUV (eSUV), delayed-phase SUV (dSUV), standardized uptake value ratios (SUVR), and the difference between early and delayed uptake (SUV_diff_) were analyzed across predefined cortical regions. Group differences were assessed using nonparametric tests, with false discovery rate (FDR) and Bonferroni corrections applied for multiple comparisons. Diagnostic performance was evaluated using receiver operating characteristic (ROC) curve analysis. *Results*: Several regional parameters demonstrated nominally significant group differences in uncorrected analyses; however, none remained statistically significant after correction for multiple comparisons. Among the evaluated metrics, SUV_diff_ demonstrated the highest diagnostic performance (sensitivity 84.2%, specificity 80.0%), followed by eSUV (68.4%, 100%) and MRI cortical volume (47.4%, 100%). Delayed-phase parameters alone showed limited discriminatory robustness despite observed group-level differences. *Conclusions*: In this exploratory cohort, SUV_diff_ showed moderate discriminatory potential between AD-MFI and MCI. However, given the small sample size and multiplicity of comparisons, the results should be interpreted as hypothesis-generating. Larger, prospective studies are required to determine the reproducibility and clinical utility of dual-phase semiquantitative parameters.

## 1. Introduction

Alzheimer’s disease (AD) is the most common cause of dementia, with global prevalence expected to rise as the population ages. Epidemiological studies estimate that over 55 million people worldwide are affected by dementia, with AD accounting for 60–70% of cases [1,2,3]. Mild cognitive impairment (MCI) represents an intermediate stage between normal aging and AD, characterized by subtle cognitive decline that does not significantly interfere with daily activities. MCI affects approximately 10–15% of individuals aged ≥ 65 years, with an annual conversion rate to AD of 10–15% [4,5,6,7].

Although individuals with MCI are at increased risk of progressing to AD, not all cases advance to dementia, underscoring the importance of early and accurate differentiation [4,8,9]. The advent of disease-modifying therapies—including anti-amyloid monoclonal antibodies approved by the Food and Drug Administration—has further underscored the clinical need for precise differentiation between early AD with mild functional impairment (AD-MFI) and MCI, as early initiation of appropriate treatment may lead to better clinical outcomes [10,11,12].

Amyloid positron emission tomography (PET) imaging has become a pivotal tool for detecting amyloid beta (Aβ) deposition, contributing to the early diagnosis of AD [13,14]. The pathophysiology of AD is defined by the accumulation of Aβ plaques and neurofibrillary tangles composed of hyperphosphorylated tau [15,16]. However, conventional amyloid PET provides only a static assessment of amyloid burden, which may limit its utility in differentiating AD-MFI from MCI. This limitation is partly due to age-related Aβ deposition in cognitively normal individuals, leading to potential false positives [14,17,18].

Prior studies have primarily relied on qualitative or conventional semiquantitative approaches, which may not adequately capture subtle cerebral blood flow (CBF) or metabolic differences between AD-MFI and MCI. Recent studies have indicated that early-phase amyloid PET can reflect changes in CBF—similar to findings reported with brain SPECT, FDG PET, and even FP-CIT PET in cases of atypical Parkinsonism—offering potential clues for improving diagnostic accuracy [19,20,21,22]. Given the clinical and prognostic significance, there is a growing need for novel imaging biomarkers with enhanced diagnostic performance [23].

This study aims to evaluate comprehensive semiquantitative parameters derived from dual-phase amyloid PET imaging to distinguish AD-MFI from MCI. By assessing their diagnostic sensitivity and specificity, the goal is to improve early diagnostic accuracy, optimize patient management, and support therapeutic decision-making in the era of emerging AD treatments.

## 2. Materials and Methods

### 2.1. Participants

In this study, a total of 24 participants were retrospectively enrolled in consecutive order between March 2024 and February 2025. Demographic information and clinical diagnoses were obtained from medical records. The inclusion criteria were: age between 55 and 90 years; clinical stages 3–4 according to the National Institute on Aging and Alzheimer’s Association (NIA-AA) criteria; a global Clinical Dementia Rating (CDR) score between 0.5 and 1.0; a brain Aβ plaque load (BAPL) score of 2 or 3; and absence of brain lesions unrelated to AD on magnetic resonance imaging (MRI). Exclusion criteria included a BAPL score of 1, a history of brain trauma, frontotemporal dementia, dementia with Lewy bodies, Huntington’s disease, or Parkinson’s disease. All participants underwent dual-phase amyloid PET and were classified into AD-MFI and MCI based on the clinical diagnosis.

The term ‘AD-MFI’ is used operationally in this study to describe individuals classified as NIA-AA stages 3–4 with mild functional impairment (CDR 0.5–1.0) and positive amyloid biomarkers (BAPL 2–3). It does not represent a formally established diagnostic category but was defined for analytical grouping purposes.

Due to strict inclusion criteria (dual-phase PET availability, NIA-AA stage 3–4 classification, positive amyloid status, and MRI compatibility), the final eligible cohort was limited. Therefore, this investigation was structured as an exploratory pilot feasibility study intended for hypothesis generation rather than confirmatory diagnostic validation.

This retrospective study was approved by the Institutional Review Board of our institution (IRB No. 2023-09-007 on 27 June 2025), which waived the requirement for informed consent due to the retrospective design of the study. The study was conducted in accordance with the ethical standards of the institutional research committee and the Declaration of Helsinki (1975, revised 2013).

### 2.2. Image Acquisition

PET/CT scans were performed using a GE Discovery MI Gen 2 (GE Healthcare, Waukesha, WI, USA). The dual-phase amyloid PET/CT protocol consisted of two phases: an early-phase and a delayed-phase. The early-phase, which represents CBF, was acquired approximately 2.7 ± 1.6 min (median 2.0 min, range 1.0–6.6 min) after the intravenous injection of 296 MBq (8 mCi) of F-18 florbetaben as a fixed dose. The delayed-phase, which reflects Aβ deposition, was acquired 90 min post-injection. Both the early- and delayed-phases had the same acquisition duration of 20 min [24].

Reconstructed PET/CT images were obtained with a 256 × 256 matrix size and 0.97 mm pixel spacing. GE’s Q.Clear (QCFX), a regularized iterative reconstruction algorithm run to convergence without preset iterations or subsets, was used for image reconstruction.

Brain MRI scans were performed using a Siemens Magnetom Skyra (Siemens Healthineers, Erlangen, Germany) with an MPRAGE 3D-T1-weighted imaging sequence. The imaging parameters were as follows: repetition time (TR) 2300 milliseconds (ms), echo time (TE) 2.98 ms, inversion time (TI) 900 ms, matrix size of 256 × 256, and a slice thickness of 1 mm in the sagittal plane.

### 2.3. Quantitative Analysis

MRI and PET images were processed using FreeSurfer and PETSurfer software (version 7.4.1). Motion correction was applied to account for patient movement, followed by skull stripping to isolate brain tissue. PET images were then coregistered to MRI for precise anatomical alignment, yielding a final PET voxel size of 1.0 × 1.0 × 1.0 mm. Standardized uptake values (SUVs) were calculated for each voxel using the following formula [25]:

$$SUV=\frac{Decay-corrected\;radiotraceractivity\left(Bq\right)\;/\;(voxel\;size\;in\;{mm}^{3}\times 10^{3})(mL)}{Injected\;dose\;\left(Bq\right)\;/\;Body\;weight\;(g)}$$

where:

• Decay-corrected radiotracer activity in Bq/voxel refers to the measured radiotracer activity at the time of acquisition, corrected to the injection time using the decay correction formula:

$$A_{t_{0}}=A_{t}\times e^{\lambda (t-t_{0})}$$

where $A_{t_{0}}$ is the estimated activity at the injection time, $A_{t}$ is the measured activity at the acquisition time, *λ* is the decay constant of F-18 (*λ* = $\frac{\ln2}{109.77}{min}^{-1}$), and $t-t_{0}$ is the elapsed time in minutes.

• Voxel size is the physical volume of the voxel in cubic millimeters (mm^3^), calculated from the voxel’s dimensions.

Subsequently, cortical and subcortical analyses were conducted to assess a combined parameter representing early-phase CBF, as well as delayed-phase amyloid deposition, across subregions of the left and right frontal, parietal, temporal, posterior cingulate, and precuneus cortices. The analyses involved quantifying the mean SUV in predefined cortical and subcortical volumes of interest (VOIs) and calculating the SUV ratio (SUVR) using the pons as the reference region, which is commonly employed for normalizing amyloid deposition. These analyses were performed separately for the early phase (eSUV, eSUVR) and delayed phase (dSUV, dSUVR) of the dual-phase amyloid PET scans.

A new quantitative parameter, SUV difference (SUV_diff`_), was calculated for each VOI by subtracting dSUV from eSUV, as follows:

SUV_diff_ = *eSUV* − *dSUV*

This parameter may serve as a surrogate marker for CBF differences, analogous to R1 as reported in prior dual-phase PET studies [19].

Group analysis using a general linear model (GLM) across volumes and surfaces was performed for eSUV, dSUV, and SUV_diff_ without covariates.

### 2.4. Qualitative Analysis

Delayed-phase PET images were visually interpreted in transaxial orientation using gray scale by two experienced nuclear medicine board-certified physicians (H.J.C. and S.H.L., with 12 years and 14 years in PET reading, respectively). Cortical regions—including the lateral temporal, frontal, posterior cingulate/precuneus, and parietal cortices—were systematically evaluated by comparing gray- and white-matter uptake and scored according to RCTU and BAPL criteria. Discrepancies were resolved by consensus.

### 2.5. Statistical Analysis

Mann–Whitney U tests and Fisher’s exact tests were conducted to assess differences in sex, age, symptom duration, education, ApoE alleles, CDR, Mini-Mental State Examination (MMSE), and quantitative VOI parameters between the MCI and AD-MFI groups. Receiver operating characteristic (ROC) curve analysis was performed to determine the optimal cutoff values for quantitative parameters within the VOIs. Only quantitative parameters with an area under the curve (AUC) > 0.5 and whose ROC curves did not cross the reference line were selected. Each selected parameter was classified as positive or negative based on its optimal cutoff, which maximized sensitivity while maintaining the highest specificity. The parameters represented subregions of the left and right frontal, parietal, temporal, posterior cingulate, and precuneus cortices. A cortex was considered positive if any of its subregions exceeded the cutoff, following the principle used in visual interpretation. Sensitivity and specificity were then evaluated for distinguishing AD-MFI from MCI.

In this study, statistical significance refers to group-level differences identified using hypothesis testing (e.g., Mann–Whitney U test), whereas diagnostic significance refers to classification performance at the individual level assessed by ROC analysis (AUC, sensitivity, and specificity). Parameters demonstrating statistical group differences were further evaluated for diagnostic robustness using ROC analysis. To address multiplicity across regional comparisons, False Discovery Rate (FDR) and Bonferroni corrections were additionally applied in supplementary analyses.

Statistical analyses were conducted using IBM SPSS Statistics 22 (IBM Corp., Armonk, NY, USA), and a *p*-value < 0.05 was considered statistically significant. Additional multiple-comparison corrections (FDR and Bonferroni methods) were performed using R statistical software (version 4.5.2; R Foundation for Statistical Computing, Vienna, Austria).

## 3. Results

### 3.1. Demographics

The AD-MFI (*n* = 19) and MCI (*n* = 5) groups exhibited no statistically significant differences in sex, age, symptom duration, education, ApoE4 status, CDR scores, MMSE, and BAPL. Among the participants, 4 were male, and 20 were female. ApoE4 status was unavailable for five participants. The post-injection time interval for eSUV and dSUV was not significantly different between the groups, minimizing potential timing-related confounding effects on the qualitative or quantitative analyses of the amyloid PET images. A summarized overview of participant demographics is presented in Table 1.

### 3.2. Dual-Phase PET/CT Comparison Between AD-MFI and MCI Groups

**Figure 1:**
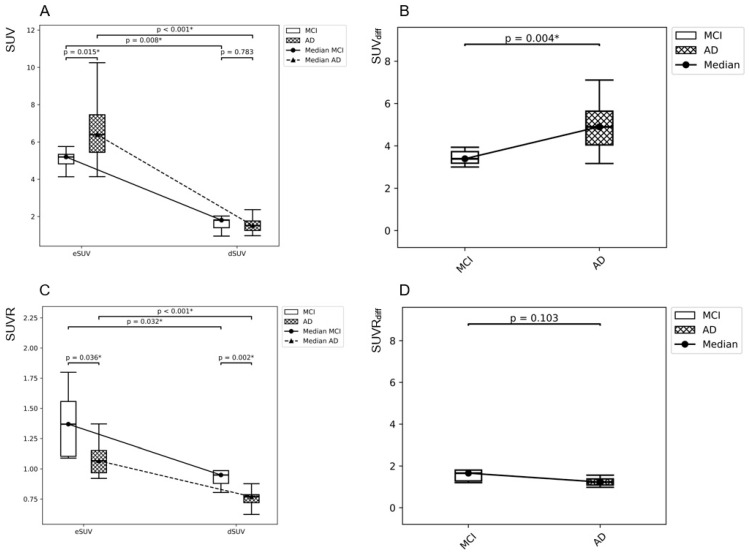
Quantitative parameters of representative brain regions showing significant differences between the AD-MFI and MCI groups in Mann–Whitney U tests. (**A**) eSUV and dSUV of the R BSTS. (**B**) SUV_diff_ of the same region. (**C**) eSUVR and dSUVR, and (**D**) SUVR_diff_ of the L SMG. Significant intergroup differences were indicated in eSUV, SUV_diff_, eSUVR, and dSUVR. No significant differences were observed in dSUV (**A**) or SUVR_diff_ (**D**). * indicates statistical significance.

The early-phase acquisition window ranged from 1.0 to 6.6 min post-injection. Although this variability may introduce measurement heterogeneity, no statistically significant intergroup difference in post-injection timing was observed. None of the whole cerebral gray matter (cbGM) quantitative parameters showed significant differences between the AD-MFI and MCI groups. Based on uncorrected Mann–Whitney U tests (*p* < 0.05), three brain regions showed significant differences in eSUV and eSUVR values, while six regions showed significant differences in dSUVR and SUV_diff_. For SUVR_diff_, only one brain region demonstrated a significant difference. Detailed statistical results are presented in Table 2 and illustrated in Figure 1.

In the initial regional analyses, several regions demonstrated nominal statistical significance (*p* < 0.05). However, after applying multiple-comparison corrections using the FDR and Bonferroni methods across all regional comparisons, none of these findings remained statistically significant. The adjusted *p*-values are provided in Supplementary Tables S1 and S2. These results indicate that the observed regional differences should be interpreted cautiously and considered exploratory, particularly given the limited sample size.

Importantly, statistical significance in these analyses reflects group-level differences between AD-MFI and MCI. Such findings do not necessarily imply diagnostic utility at the individual patient level.

### 3.3. ROC Curve Analysis of Dual-Phase PET/CT and MRI

**Figure 2:**
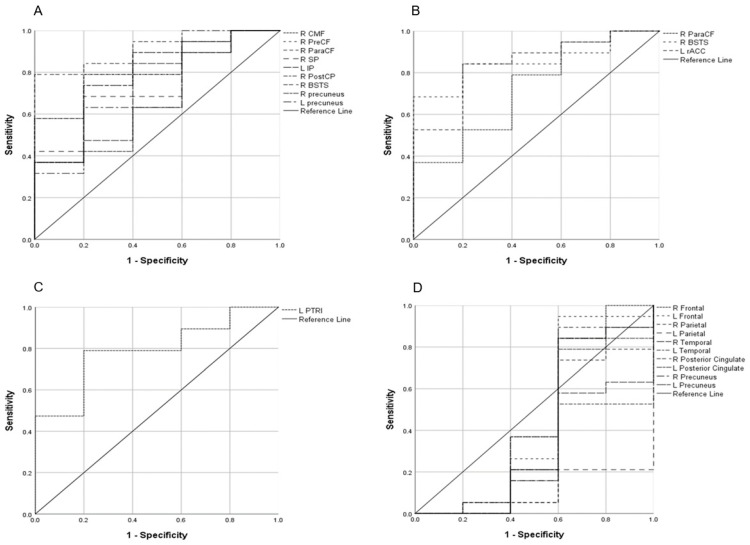
ROC curve analyses of SUV_diff_ (**A**), eSUV (**B**), and MRI cortical volume (**C**) for identifying parameters that met the selection criteria: AUC > 0.5 without crossing the reference line. The SUV_diff_ of the R BSTS (**A**) demonstrated the highest sensitivity and specificity. None of the dSUVR parameters met the predefined selection criteria; representative ROC curves for selected brain regions are shown for illustrative purposes (**D**).

ROC curve analysis was conducted to evaluate individual-level classification performance. Three eSUV regions, nine SUV_diff_ regions, and the pars triangularis of the frontal lobe for cortical volume met the selection criteria (AUC > 0.5 and ROC curve not crossing the reference line). Among these, SUV_diff_ demonstrated the highest diagnostic performance, with a sensitivity of 84.2% and specificity of 80.0%. eSUV showed a sensitivity of 68.4% and specificity of 100%, while cortical volume demonstrated lower sensitivity (47.4%) but high specificity (100%). BAPL classification alone yielded a sensitivity of 68.4% and limited specificity (20.0%). Although certain delayed-phase parameters (e.g., dSUVR in selected regions) demonstrated statistically significant group-level differences in regional comparison analyses, none achieved adequate diagnostic robustness in ROC analysis. This distinction underscores the difference between statistical group separation and clinically meaningful diagnostic discrimination. Detailed results are provided in Table 3 and Table 4 and Figure 2.

### 3.4. GLM-Based Group Analysis of Early- and Delayed-Phase PET/CT

**Figure 3:**
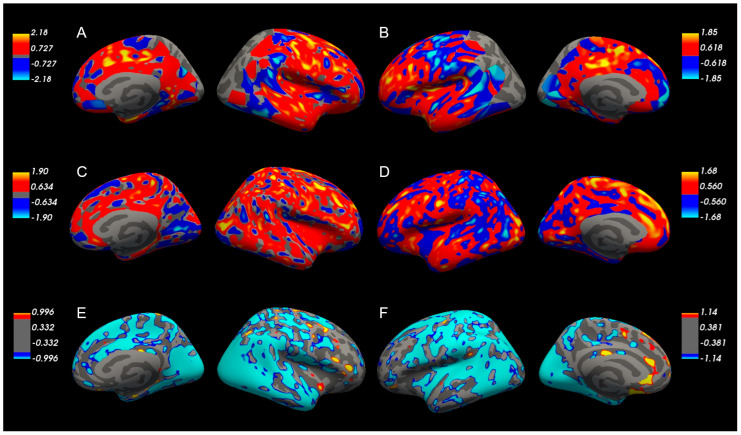
GLM-based group analysis of Z-scores for SUV parameters between the AD-MFI and MCI groups. The Z-scores represent the standardized regression coefficients derived from group contrasts in the general linear model, indicating the magnitude and direction of group differences. The Z-scores of SUV_diff_ (**A**,**B**): right and left hemispheres) and eSUV (**C**,**D**): right and left hemispheres) were higher in the AD-MFI group than in the MCI group, whereas the Z-scores of dSUV (**E**,**F**): right and left hemispheres) were lower in the AD-MFI group.

Voxel-wise general linear model (GLM) analyses were performed to visualize spatial patterns of group differences in SUV_diff_, eSUV, and dSUV metrics (Figure 3). The Z-scores represented standardized regression coefficients derived from the group contrast in the general linear model, reflecting the magnitude and direction of voxel-level differences between groups. SUV_diff_ (A, B: right and left hemispheres) demonstrated relatively widespread positive Z-score patterns in the AD-MFI group compared to the MCI group, particularly within bilateral frontal regions. eSUV (C, D: right and left hemispheres) exhibited regionally variable patterns with less extensive involvement. In contrast, dSUV (E, F: right and left hemispheres) showed comparatively localized differences.

These voxel-wise visualizations represent exploratory group-level patterns and were not corrected for multiple comparisons. Therefore, they should be interpreted as descriptive illustrations of spatial trends rather than definitive statistical evidence.

## 4. Discussion

In this exploratory pilot study, we evaluated semiquantitative parameters derived from dual-phase amyloid PET/CT to differentiate individuals operationally classified as AD-MFI from those with MCI. Although several regional parameters demonstrated statistically significant group-level differences in uncorrected analyses, only selected early-phase and SUV_diff_ parameters showed moderate diagnostic performance in ROC analysis. After applying multiple-comparison corrections (FDR and Bonferroni methods), none of the previously nominally significant regional findings remained statistically significant. These results underscore the importance of accounting for multiplicity in regional neuroimaging analyses and indicate that the observed differences should be interpreted as exploratory rather than confirmatory, particularly given the limited sample size.

Notably, certain delayed-phase SUV and SUVR parameters in selected cortical regions demonstrated statistically significant intergroup differences in region-wise analyses based on surface-based cortical parcellation and volume-based subcortical segmentation using FreeSurfer (v7.4.1). However, these parameters did not demonstrate adequate discriminatory performance in ROC analysis. This distinction highlights the conceptual difference between statistical significance (group-level separation) and diagnostic significance (individual-level classification performance). A parameter may differ significantly between groups yet lack sufficient sensitivity and specificity for reliable clinical discrimination.

Previous studies have reported high sensitivity (up to 90%) and specificity (approximately 80%) for amyloid PET in diagnosing AD; however, specificity decreases substantially when differentiating AD from MCI [14]. Similarly, while amyloid PET demonstrates high sensitivity (84–96%) for predicting MCI-to-AD conversion, specificity remains modest (42–62%) [14,18]. These findings illustrate the inherent limitations of conventional amyloid PET in distinguishing early AD from MCI, potentially due to age-related Aβ deposition in cognitively normal individuals and overlapping pathological burdens across disease stages.

Emerging studies of dual-phase amyloid PET/CT have reported associations between CBF–related signals and amyloid burden in individuals clinically classified as early AD; however, most evidence remains cross-sectional rather than longitudinal [26,27]. In the present study, eSUV values were relatively higher and dSUV values relatively lower in the AD-MFI group compared with the MCI group. This pattern of comparatively increased early-phase uptake and decreased delayed-phase uptake may reflect alterations in perfusion dynamics accompanying amyloid accumulation. Nevertheless, in the absence of direct perfusion validation (e.g., arterial spin labeling MRI or FDG PET correlation), SUV_diff_ should be regarded as a perfusion-related proxy rather than a validated physiological biomarker.

Unlike many prior investigations that focused primarily on SUVR, we compared both SUV and SUVR metrics to more directly evaluate perfusion-related and amyloid-related components. Although dual-phase amyloid PET/CT has been proposed to simultaneously assess CBF and amyloid deposition, many previous studies relied on kinetic modeling approaches requiring dedicated software and complex acquisition protocols that are not routinely available in clinical practice [19,27,28,29,30,31].

To address these practical limitations, we introduced SUV_diff_, defined as the difference between eSUV and dSUV, as a simplified metric reflecting the dynamic interplay between perfusion-related uptake and delayed amyloid retention. Given that R1 (a CBF surrogate) has demonstrated utility in differentiating early AD from MCI, SUV_diff_ may represent a feasible, software-independent alternative derived from routinely calculated SUVs. In uncorrected analyses, multiple regions demonstrated group differences in eSUV, eSUVR, SUVR_diff_, and SUV_diff_, with SUV_diff_ showing the most extensive spatial involvement and the highest apparent diagnostic performance (sensitivity 84.2%, specificity 80.0%). However, these findings did not remain statistically significant after correction for multiple comparisons and therefore should be interpreted cautiously.

Within the constraints of this cohort, combining early-phase perfusion-related information with delayed-phase amyloid burden into a simple difference metric may provide an incremental discriminatory signal compared with delayed-phase measures alone. Unlike kinetic modeling approaches that require dedicated software and complex acquisition protocols, SUV_diff_ is derived directly from routinely calculated SUVs, potentially increasing its feasibility in clinical practice. Nevertheless, feasibility does not equate to validation. Larger, adequately powered studies are required to determine whether SUV_diff_ provides reproducible and clinically meaningful incremental value.

This study has several limitations. First, it was a retrospective, single-center analysis with a small sample size (*n* = 24) and unequal group distribution (MCI, *n* = 5; AD-MFI, *n* = 19), limiting statistical power and generalizability. Second, the predominance of female participants (male:female = 4:20) may introduce sex-related bias. Third, the absence of cognitively normal controls precluded evaluation of SUV_diff_ in relation to amyloid-negative individuals. Fourth, the cross-sectional design prevents inference regarding longitudinal progression, causality, or prognostic value. Diagnostic categories such as AD-MFI and MCI likely encompass neurobiological heterogeneity, and subgroup analyses were not statistically feasible.

Beyond these conceptual limitations, several technical considerations should also be noted. The early-phase acquisition window demonstrated variability (1.0–6.6 min post-injection), which may have introduced measurement heterogeneity despite the absence of statistically significant intergroup timing differences. Furthermore, SUV_diff_ was interpreted as a potential indicator of perfusion-related changes rather than a validated surrogate of cerebral blood flow, and its physiological correspondence requires confirmation against established perfusion imaging modalities. Although FDR and Bonferroni corrections were applied in supplementary analyses, the large number of regional comparisons in a small sample increases the risk of both type I and type II error; therefore, findings should be interpreted cautiously. In addition, demographic comparisons were constrained by the small MCI subgroup size, limiting statistical power for between-group demographic analyses. Apolipoprotein E (ApoE4) genotype data were unavailable for a subset of participants, which may have influenced the interpretability of genetic risk stratification. Voxel-wise general linear model visualizations were intended as illustrative exploratory maps rather than definitive statistical evidence and should be interpreted cautiously, given the limited sample size.

Accordingly, the present findings should be regarded as preliminary and hypothesis-generating rather than definitive evidence. The primary objective was to evaluate the feasibility and preliminary discriminatory signal of SUV_diff_ derived from routine dual-phase PET data without kinetic modeling software, rather than to establish definitive clinical diagnostic standards. Future prospective, multicenter studies with larger and more demographically and clinically diverse cohorts will be necessary to confirm reproducibility, establish clinical relevance, and evaluate stage- and subtype-specific SUV_diff_ patterns. In addition, because the present investigation employed a cross-sectional design, no temporal, causal, or prognostic inferences regarding disease progression or clinical outcomes can be drawn. Longitudinal validation will be required to determine whether SUV_diff_ demonstrates predictive value over time.

## 5. Conclusions

Accurate differentiation between early AD and MCI is important for clinical management and timely consideration of therapeutic strategies. Within the constraints of this preliminary retrospective and exploratory pilot study, certain semiquantitative parameters derived from dual-phase amyloid PET/CT demonstrated nominal group-level differences between individuals classified as AD-MFI and those with MCI. Among the evaluated metrics, SUV_diff_ exhibited the highest diagnostic performance in ROC analysis.

However, regional findings did not remain statistically significant after correction for multiple comparisons and delayed-phase parameters alone showed limited discriminatory robustness despite statistical group differences. These results underscore the conceptual distinction between statistical significance and clinically meaningful diagnostic discrimination.

However, given the limited sample size, sex imbalance, single-center design, and potential within-group heterogeneity, these findings should be interpreted cautiously. Further validation through larger, prospective, and methodologically diverse investigations—including longitudinal and physiologically validated analyses—is necessary to determine whether SUV_diff_ provides reproducible and clinically meaningful incremental value in differentiating early Alzheimer’s disease from MCI.

## Figures and Tables

**Table 1:** Demographics of MCI and AD-MFI Groups.

Characteristics		AD-MFI (*n* = 19)	MCI (*n* = 5)	*p*
Sex, M/F		4 (21.1)/15 (78.9)	0 (0.0)/5 (100.0)	0.489
Age, years		75.0 ± 8.6	75.2 ± 6.3	0.945
Sx Duration, years		4.8 ± 1.7	5.3 ± 4.0	0.684
Education, years		8.7 ± 4.6	10.8 ± 3.7	0.354
ApoE4				0.411
	0	5 (26.3)	2 (40.0)	
	1	6 (31.6)	2 (40.0)	
	2	4 (21.1)	0 (0.0)	
	N/A	4 (21.1)	1 (20.0)	
CDR				0.130 *
	0.5	11 (57.9)	5 (100.0)	
	1	8 (42.1)	0 (0.0)	
MMSE		20 ± 4	22 ± 2	0.297
BAPL				1.000 *
	2	6 (31.6)	1 (20.0)	
	3	13 (68.4)	4 (80.0)	
Post-Injection Time Interval, min				
	eSUV	2.9 ± 1.7	2.1 ± 1.1	0.534
	dSUV	90.4 ± 5.4	87.9 ± 2.5	0.208

Mean ± standard deviation, or number (percentage). * Fisher’s Exact Test; AD, Alzheimer’s disease; AD-MFI, early AD with mild functional impairment; BAPL, brain amyloid plaque load; CDR, clinical dementia rating; dSUV, SUV in the delayed-phase; eSUV, SUV in the early-phase; F, female; M, male; MCI, mild cognitive impairment; N/A, not available; SUV, standardized uptake value; Sx, symptom.

**Table 2:** Comparison of Dual-phase PET/CT of AD-MFI and MCI groups.

Parameters	Brain Regions	AD-MFI (*n* = 19)	MCI (*n* = 5)	*p*
eSUV				
	R cbGM	5.132 ± 1.430	4.489 ± 0.878	0.446
	L cbGM	5.105 ± 1.341	4.435 ± 0.657	0.331
	R BSTS	6.659 ± 1.642	5.049 ± 0.611	0.015 *
	R PHG	3.139 ± 1.489	1.906 ± 1.325	0.036 *
	L rACC	4.697 ± 1.684	3.119 ± 0.605	0.019 *
eSUVR				
	R cbGM	1.131 ± 0.191	1.329 ± 0.448	0.679
	L cbGM	1.127 ± 0.172	1.307 ± 0.373	0.367
	L HPC	0.616 ± 0.123	0.765 ± 0.065	0.005 *
	L BSTS	1.263 ± 0.158	1.536 ± 0.389	0.044 *
	L SMG	1.096 ± 0.162	1.384 ± 0.303	0.036 *
dSUV				
	R cbGM	1.272 ± 0.326	1.275 ± 0.342	0.783
	L cbGM	1.279 ± 0.326	1.267 ± 0.272	0.945
	R BSTS	1.581 ± 0.440	1.601 ± 0.429	0.783
dSUVR				
	R cbGM	0.763 ± 0.092	0.868 ± 0.190	0.446
	L cbGM	0.766 ± 0.083	0.867 ± 0.135	0.160
	L PreCF	0.694 ± 0.086	0.822 ± 0.097	0.019 *
	L SP	0.732 ± 0.096	0.891 ± 0.075	0.001 *
	L SMG	0.762 ± 0.094	0.957 ± 0.135	0.002 *
	L PostCP	0.689 ± 0.085	0.822 ± 0.106	0.024 *
	L FFG	0.746 ± 0.091	0.844 ± 0.078	0.036 *
	L TTG	0.662 ± 0.095	0.854 ± 0.162	0.007 *
SUV_diff_				
	R cbGM	3.859 ± 1.189	3.215 ± 0.547	0.235
	L cbGM	3.826 ± 1.095	3.167 ± 0.389	0.235
	R CMF	4.679 ± 1.534	3.060 ± 0.889	0.036 *
	R PTRI	4.048 ± 1.639	2.740 ± 0.815	0.044 *
	R PreCF	4.755 ± 1.571	3.247 ± 0.882	0.036 *
	R BSTS	5.078 ± 1.397	3.448 ± 0.384	0.004 *
	R PHG	2.130 ± 1.352	0.975 ± 1.233	0.030 *
	L rACC	3.264 ± 1.340	1.946 ± 0.409	0.007 *
SUVR_diff_				
	R cbGM	0.931 ± 0.125	1.178 ± 0.495	0.208
	L cbGM	0.980 ± 0.129	1.225 ± 0.589	0.731
	L SMG	1.310 ± 0.325	1.793 ± 0.727	0.103
	L HPC	0.653 ± 0.202	0.901 ± 0.182	0.012 *
Volume, mL				
	R cbGM	217.609 ± 20.884	215.030 ± 12.844	0.972
	L cbGM	217.525 ± 19.979	214.921 ± 13.358	0.972
Thickness, mm				
	L POP	2.288 ± 0.184	2.332 ± 0.215	0.594
	L PTRI	2.501 ± 0.300	2.369 ± 0.392	0.227
	L LOF	2.751 ± 0.205	2.839 ± 0.281	0.355
	L MOF	2.708 ± 0.221	2.792 ± 0.181	0.434
	R PostCP	1.801 ± 0.134	1.797 ± 0.115	0.859
	L TP	3.503 ± 0.301	3.401 ± 0.380	0.803
	L cACC	2.713 ± 0.392	2.759 ± 0.331	0.859

Mean ± standard deviation. * *p* < 0.05; AD, Alzheimer’s disease; AD-MFI, early AD with mild functional impairment; BSTS, the banks of the superior temporal sulcus of the lateral temporal cortices; cACC, caudal anterior cingulate cortex; cbGM, cerebral gray matter; CMF, caudal middle frontal; dSUV, SUV in the delayed-phase; dSUVR, SUVR in the delayed-phase; eSUV, SUV in the early-phase; eSUVR, SUVR in the early-phase; FFG, the fusiform gyrus of lateral temporal lobe; HPC, hippocampus; L, left; LOF, lateral orbitofrontal; MCI, mild cognitive impairment; MOF, medial orbitofrontal; ParaCF, paracentral frontal; PHG, the parahippocampal gyrus in the medial temporal lobe; POP, the pars opercularis of the frontal lobe; PostCP, postcentral gyri of the parietal lobe; PreCF, precentral frontal; PTRI, pars triangularis of the frontal lobe; R, right; rACC, rostral anterior cingulate cortex; SMG, supramarginal parietal cortex; SP, superior parietal cortex; SUV, standardized uptake value; SUV_diff_ = eSUV − dSUV; SUVR, SUV ratio; TP, temporal pole; TTG, transverse temporal cortex.

**Table 3:** Selected Brain Regions and Cutoffs for ROC Curve Analysis.

Parameters	Selected Brain Regions	Cutoff	AUC	*p*
eSUV				
	R ParaCF	6.108	0.726	0.126
	R BSTS	5.798	0.853	0.017 *
	L rACC	3.869	0.842	0.093
SUV_diff_				
	R CMF	4.112	0.811	0.036 *
	R PreCF	4.417	0.811	0.036 *
	R ParaCF	4.818	0.779	0.060
	R SP	3.896	0.747	0.095
	L IP	4.043	0.716	0.145
	R PostCP	4.744	0.789	0.051
	R BSTS	3.937	0.905	0.006 *
	R precuneus	3.911	0.674	0.241
	L precuneus	3.861	0.716	0.145
Volume, mL				
	L PTRI	3.471	0.789	0.051

* *p* < 0.05; AUC, area under the curve; BSTS, the banks of the superior temporal sulcus of the lateral temporal cortices; CMF, caudal middle frontal; dSUV, SUV in the delayed-phase; eSUV, SUV in the early-phase; IP, inferior parietal; L, left; ParaCF, paracentral frontal; PostCP, postcentral gyri of the parietal lobe; PreCF, precentral frontal; PTRI, pars triangularis of the frontal lobe; R, right; rACC, rostral anterior cingulate cortex; SP, superior parietal; SUV, standardized uptake value; SUV_diff_ = eSUV − dSUV; SUVR, SUV ratio.

**Table 4:** Sensitivity and Specificity of Quantitative Parameters for distinguishing AD-MFI from MCI.

Parameters		AD-MFI (*n* = 19)	MCI (*n* = 5)	Total (*n* = 24)
BAPL				
	3	13 (68.4)	4 (80.0)	17
	2	6 (31.6)	1 (20.0)	7
eSUV				
	+ *	13 (68.4)	0 (0.0)	13
	- ^†^	6 (31.6)	5 (100.0)	11
SUV_diff_				
	+ *	16 (84.2)	1 (20.0)	17
	- ^†^	3 (15.8)	4 (80.0)	7
Volume of L PTRI				
	+ *	9 (47.4)	0 (0.0)	19
	- ^†^	10 (52.6)	5 (100.0)	9

Number (percentage). AD, Alzheimer’s disease; AD-MFI, early AD with mild functional impairment; BAPL, brain amyloid β plaque load; dSUV, SUV in the delayed-phase; eSUV, SUV in the early-phase; MCI, mild cognitive impairment; PTRI, pars triangularis of the frontal lobe; SUV, standardized uptake value; SUV_diff_ = eSUV − dSUV. * Classified as positive if any brain subregion exceeds the cutoff. ^†^ Classified as negative only if all brain subregions are below the cutoff.

## Data Availability

The datasets used and/or analyzed during the current study are available from the corresponding author on reasonable request.

## Supplementary Materials

The following supporting information can be downloaded at: https://www.mdpi.com/article/10.3390/medicina62030529/s1, Supplementary Table S1. Corrected Comparison of Dual-Phase PET/CT Parameters Between the AD-MFI and MCI Groups; Supplementary Table S2. Corrected Selected Brain Regions and Cutoff Values for ROC Curve Analysis.
