# Supplementary material for: Differentiating Early Alzheimer’s Disease from MCI Using Comprehensive Semiquantitative Parameters in Dual-Phase Amyloid PET: A Pilot Study
Source: Medicina (Kaunas). 2026 Mar 12;62(3):529. doi: 10.3390/medicina62030529 (PMC13027799; doi:10.3390/medicina62030529)
Supplement: Supplementary file 1 [file medicina-62-00529-s001.zip › medicina-4163741-supplementary.pdf]

Supplementary Table S1. Corrected Comparison of Dual-Phase PET/CT Parameters Between the AD-MFI and MCI Groups

Parameters	Brain Regions	AD-MFI (n=19)	MCI (n=5)	*P*	FDR-adjusted *P*	Bonferroni-adjusted *P*
eSUV						
	R cbGM	5.132±1.430	4.489±0.878	0.446	0.703	>0.999
	L cbGM	5.105±1.341	4.435±0.657	0.331	0.703	>0.999
	R BSTS	6.659±1.642	5.049±0.611	0.015*	0.703	>0.999
	R PHG	3.139±1.489	1.906±1.325	0.036*	0.703	>0.999
	L rACC	4.697±1.684	3.119±0.605	0.019*	0.703	>0.999
eSUVR						
	R cbGM	1.131±0.191	1.329±0.448	0.679	0.908	>0.999
	L cbGM	1.127±0.172	1.307±0.373	0.367	0.869	>0.999
	L HPC	0.616±0.123	0.765±0.065	0.005*	0.741	0.750
	L BSTS	1.263±0.158	1.536±0.389	0.044*	0.741	>0.999
	L SMG	1.096±0.162	1.384±0.303	0.036*	0.741	>0.999
dSUV						
	R cbGM	1.272±0.326	1.275±0.342	0.783	>0.999	>0.999
	L cbGM	1.279±0.326	1.267±0.272	0.945	>0.999	>0.999
	R BSTS	1.581±0.440	1.601±0.429	0.783	>0.999	>0.999
dSUVR						
	R cbGM	0.763±0.092	0.868±0.190	0.446	0.626	>0.999
	L cbGM	0.766±0.083	0.867±0.135	0.160	0.455	>0.999
	L PreCF	0.694±0.086	0.822±0.973	0.019*	0.193	>0.999
	L SP	0.732±0.096	0.891±0.075	0.001*	0.132	0.313
	L SMG	0.762±0.094	0.957±0.135	0.002*	0.132	0.393
	L PostCP	0.689±0.085	0.822±0.106	0.024*	0.193	>0.999
	L FFG	0.746±0.091	0.844±0.078	0.036*	0.216	>0.999
	L TTG	0.662±0.095	0.854±0.162	0.007*	0.132	0.924
SUV_{diff}						

	R cbGM	3.859±1.189	3.215±0.547	0.235		
	L cbGM	3.826±1.095	3.167±0.389	0.235		
	R CMF	4.679±1.534	3.060±0.889	0.036*		
	R PTRI	4.048±1.639	2.740±0.815	0.044*		
	R PreCF	4.755±1.571	3.247±0.882	0.036*		
	R BSTS	5.078±1.397	3.448±0.384	0.004*		
	R PHG	2.130±1.352	0.975±1.233	0.030*		
	L rACC	3.264±1.340	1.946±0.409	0.007*		
$SUVR_{diff}$						
	R cbGM	0.931±0.125	1.178±0.495	0.208		
	L cbGM	0.980±0.129	1.225±0.589	0.731		
	L SMG	1.310±0.325	1.793±0.727	0.103		
	L HPC	0.653±0.202	0.901±0.182	0.012*		
Volume, mL						
	R cbGM	217.609±20.884	215.030±12.844	0.972	>0.999	>0.999
	L cbGM	217.525±19.979	214.921±13.358	0.972	>0.999	>0.999
Thickness, mm						
	L POP	2.288±0.184	2.332±0.215	0.594	>0.999	>0.999
	L PTRI	2.501±0.300	2.369±0.392	0.227	>0.999	>0.999
	L LOF	2.751±0.205	2.839±0.281	0.355	>0.999	>0.999
	L MOF	2.708±0.221	2.792±0.181	0.434	>0.999	>0.999
	R PostCP	1.801±0.134	1.797±0.115	0.859	>0.999	>0.999
	L TP	3.503±0.301	3.401±0.380	0.803	>0.999	>0.999
	L cACC	2.713±0.392	2.759±0.331	0.859	>0.999	>0.999

Mean ± standard deviation.

*P < 0.05

AD, Alzheimer's disease; AD-MFI, early AD with mild functional impairment; BSTS, the banks of the superior temporal sulcus of the lateral temporal cortices; cACC, caudal anterior cingulate cortex; cbGM, cerebral gray matter; CMF, caudal middle frontal; dSUV, SUV in the delayed-phase; dSUVR, SUVR in the delayed-phase; eSUV, SUV in the early-phase; eSUVR, SUVR in the early-phase; FDR, false discovery rate; FFG, the fusiform gyrus of lateral temporal lobe; HPC, hippocampus; L, left; LOF, lateral orbitofrontal; MCI, mild cognitive impairment; MOF, medial orbitofrontal; ParaCF, paracentral frontal; PHG, the parahippocampal gyrus in the medial temporal lobe; POP, the pars opercularis of the frontal

lobe; PostCP, postcentral gyri of the parietal lobe; PreCF, precentral frontal; PTRI, pars triangularis of the frontal lobe; R, right; rACC, rostral anterior cingulate cortex; SMG, supramarginal parietal cortex; SP, superior parietal cortex; SUV, standardized uptake value; SUVdiff = eSUV - dSUV; SUVR, SUV ratio; TP, temporal pole; TTG, transverse temporal cortex

Supplementary Table S2. Corrected Selected Brain Regions and Cutoff Values for ROC Curve Analysis

Parameters	Selected Brain Regions	Cutoff	*AUC*	*P*	FDR-adjusted *P*	Bonferroni-adjusted *P*
eSUV						
	R ParaCF	6.108	0.726	0.126	0.361	>0.999
	R BSTS	5.798	0.853	0.017*	0.361	0.678
	L rACC	3.869	0.842	0.093	0.361	0.854
SUV_{diff}						
	R CMF	4.112	0.811	0.036*	0.230	>0.999
	R PreCF	4.417	0.811	0.036*	0.230	>0.999
	R ParaCF	4.818	0.779	0.060	0.230	>0.999
	R SP	3.896	0.747	0.095	0.230	>0.999
	L IP	4.043	0.716	0.145	0.230	>0.999
	R PostCP	4.744	0.789	0.051	0.230	>0.999
	R BSTS	3.937	0.905	0.006*	0.157	0.174
	R precuneus	3.911	0.674	0.241	0.245	>0.999
	L precuneus	3.861	0.716	0.145	0.230	>0.999
Volume, mL						
	L PTRI	3.471	0.789	0.051	0.706	>0.999

*P < 0.05

AUC, area under the curve; BSTS, the banks of the superior temporal sulcus of the lateral temporal cortices; CMF, caudal middle frontal; dSUV, SUV in the delayed-phase; eSUV, SUV in the early-phase;

IP, inferior parietal; L, left; ParaCF, paracentral frontal; PostCP, postcentral gyri of the parietal lobe; PreCF, precentral frontal; PTRI, pars triangularis of the frontal lobe; R, right; rACC, rostral anterior cingulate cortex; SP, superior parietal; SUV, standardized uptake value; SUV_{diff} = eSUV - dSUV; SUVR, SUV ratio
